# Supplementary material for: MetaQTL: a package of new computational methods for the meta-analysis of QTL mapping experiments
Source: BMC Bioinformatics. 2007 Feb 8;8:49. doi: 10.1186/1471-2105-8-49 (PMC1808479; doi:10.1186/1471-2105-8-49)
Supplement: Additional File 7 — MetaQTL Package : jar file and tutorial. This Zip archive contains both the MetaQTL JAR file and the files of the tutorial. [file 1471-2105-8-49-S7.zip › org.inra.metaqtl/doc/index-files/index-7.html]

 
 
 
 
 
 
G-Index
 


 

 
function windowTitle()
{
    parent.document.title="G-Index";
}
 
 
 

 

 


 
   
  
 
 
 
   
 
   
          Overview     
        Package   
        Class   
        Use   
          Tree     
          Deprecated     
       Index    
          Help     
   
 
 
  
 
 
 

 
  
   PREV LETTER   
   NEXT LETTER    
  
    FRAMES     
   NO FRAMES     
  
    All Classes  ');
  }
  //-->
 
 
    All Classes  
 


  
 
 
  
 

 A   B   C   D   E   F   G   H   I   K   L   M   N   O   P   Q   R   S   T   U   V   W   X   Y   Z   
    
 G  
 
   G   - 
Variable in class org.metaqtl.util. OffspringManager.HaldaneWaddingtonProportion 
  
   G_coeff   - 
Static variable in class org.metaqtl.util. OffspringManager.HaldaneWaddingtonProportion 
  
   GAMCOF   - 
Static variable in class org.metaqtl.numrec. NumericalUtilities 
  
   gammln(double)   - 
Static method in class org.metaqtl.numrec. NumericalUtilities 
  
   gammp(double, double)   - 
Static method in class org.metaqtl.numrec. NumericalUtilities 
  
   gauss(double)   - 
Static method in class org.metaqtl.numrec. NumericalUtilities 
 This routine returns the value at x of the normal
 centered gaussian density.
   gcf(double, double)   - 
Static method in class org.metaqtl.numrec. NumericalUtilities 
  
   gen   - 
Variable in class org.metaqtl. Qtl.QTLCross 
 The number of generations.
   gen   - 
Static variable in class org.thalia.bio.util. BioCrossTypes 
  
   generalHelp()   - 
Static method in class org.metaqtl.main. MetaMain 
  
   generalUsage()   - 
Static method in class org.metaqtl.main. MetaMain 
  
   GeneticMap   - Class in  org.thalia.bio.entity  Class Description Here   GeneticMap()   - 
Constructor for class org.thalia.bio.entity. GeneticMap 
  
   GeneticMap(String, IBioEntity)   - 
Constructor for class org.thalia.bio.entity. GeneticMap 
  
   GeneticMap(String)   - 
Constructor for class org.thalia.bio.entity. GeneticMap 
  
   GeneticMapBean   - Class in  org.thalia.bio.entity.bean  Class Description Here   GeneticMapBean()   - 
Constructor for class org.thalia.bio.entity.bean. GeneticMapBean 
  
   GeneticMapBeanAdapter   - Class in  org.thalia.bio.entity.adapter  Class Description Here   GeneticMapBeanAdapter()   - 
Constructor for class org.thalia.bio.entity.adapter. GeneticMapBeanAdapter 
  
   GeneticMapProperties   - Class in  org.metaqtl.util  General comments must be added here.   GeneticMapProperties()   - 
Constructor for class org.metaqtl.util. GeneticMapProperties 
  
   GENOME   - 
Static variable in interface org.thalia.bio. IBioEntityTypes 
 The integer code for Genome entity
   getAdjacentTable()   - 
Method in class org.metaqtl. ChromCluster.ClusterGraph 
  
   getAllele(String)   - 
Method in class org.thalia.bio.entity. AlleleContainer 
  
   getAllele(String)   - 
Method in interface org.thalia.bio. IBioLocus 
  
   getAlleleNumber()   - 
Method in class org.thalia.bio.entity. AlleleContainer 
  
   getAlleleNumber()   - 
Method in interface org.thalia.bio. IBioLocus 
  
   getBestClustering(int)   - 
Method in class org.metaqtl. MetaQtlResult 
 This methods returns the best models for each criterion
 defined to select the optimal number of clusters per
 trait.
   getBestModel(int)   - 
Method in class org.metaqtl. MetaQtlAnalysis 
 This methods returns the best models for each criterion
 defined to select the optimal number of clusters per
 chromosome per trait.
   getBestResult(EMResult[], int)   - 
Static method in class org.metaqtl. EMCriteria 
 For the given criterion  criterion  this methods
 looks into the array of   EMResult   and find the model
 which is optimal.
   getBestResult(EMResult[], int, int)   - 
Static method in class org.metaqtl. EMCriteria 
  
   getBestResult(EMResult[], String)   - 
Static method in class org.metaqtl. EMCriteria 
  
   getBestResult(EMResult[], int, String)   - 
Static method in class org.metaqtl. EMCriteria 
  
   getBioAdapter()   - 
Method in class org.thalia.bio.entity. BioEntityCollection 
  
   getBioAdapter()   - 
Method in class org.thalia.bio.entity. GeneticMap 
  
   getBioAdapter()   - 
Method in class org.thalia.bio.entity. Individual 
  
   getBioAdapter()   - 
Method in class org.thalia.bio.entity. LGroup 
  
   getBioAdapter()   - 
Method in class org.thalia.bio.entity. Marker 
  
   getBioAdapter()   - 
Method in class org.thalia.bio.entity. Ontology 
  
   getBioAdapter()   - 
Method in class org.thalia.bio.entity. OntologyTerm 
  
   getBioAdapter()   - 
Method in class org.thalia.bio.entity. Population 
  
   getBioAdapter()   - 
Method in class org.thalia.bio.entity. QTL 
  
   getBioAdapter()   - 
Method in interface org.thalia.bio. IBioAdaptable 
  
   getCard()   - 
Method in class org.metaqtl. TreeNode 
  
   getCentroid(double[], double[])   - 
Method in class org.metaqtl. TreeNode 
  
   getChildren()   - 
Method in class org.metaqtl. TreeNode 
  
   getChildrenTerm()   - 
Method in class org.thalia.bio.entity. OntologyTerm 
  
   getChildrenTerm()   - 
Method in interface org.thalia.bio. IBioOntologyTerm 
 Returns the children if the term has chilren,
 null otherwise.
   getChromAxe()   - 
Method in class org.metaqtl.graph. ChromLayer 
 Return the axe of the chromosome.
   getChromAxe()   - 
Method in class org.metaqtl.graph. ChromShape 
  
   getChromAxe()   - 
Method in class org.metaqtl.graph. QtlPartitionLayer 
  
   getChromByNames(Chromosome[], String[])   - 
Static method in class org.metaqtl.main. MetaMain 
  
   getChromGraph(Chromosome[], boolean, int, IBioOntology, MetaQtlAnalysis)   - 
Static method in class org.metaqtl.main. MetaMain 
  
   getChromIdx(String)   - 
Method in class org.metaqtl. MetaQtlAnalysis 
  
   getChromNames()   - 
Method in class org.metaqtl. MetaQtlModel 
 Returns the names of the chromosomes.
   getChromosomeName(String)   - 
Method in class org.metaqtl. MapCluster 
 This method returns the name of the chromosome
 from the given name.
   getChromWidth()   - 
Method in class org.metaqtl.graph. ChromLayer 
  
   getChromWidth()   - 
Method in class org.metaqtl.graph. ChromShape 
  
   getClusterByNames(ChromCluster[], String[])   - 
Static method in class org.metaqtl.main. MetaMain 
  
   getClustering(int)   - 
Method in class org.metaqtl. MetaQtlResult 
 Returns the Qtl clustering result with  k 
 clusters.
   getClusterMembers()   - 
Method in class org.metaqtl. ChromCluster 
 This method returns the members of the cluster as an
 array of  Chromosomes .
   getClusters()   - 
Method in class org.metaqtl. MapCluster 
 Returns the chromosome clusters.
   getCMrkTotalNumbers()   - 
Method in class org.metaqtl. ChromCluster 
  
   getCommonMarkerPaths(ChromLayer)   - 
Method in class org.metaqtl.graph. ChromLayer 
  
   getCommonMarkerPoints(ChromLayer)   - 
Method in class org.metaqtl.graph. ChromLayer 
  
   getCopy()   - 
Method in class org.metaqtl. Qtl 
 Returns a copy of the QTL.
   getCopy()   - 
Method in class org.metaqtl. Qtl.QTLCross 
  
   getCopy()   - 
Method in class org.metaqtl. Qtl.QTLProj 
  
   getCopy()   - 
Method in class org.metaqtl. Qtl.QTLStats 
  
   getCopy()   - 
Method in class org.metaqtl. Qtl.QTLSupport 
  
   getCopy()   - 
Method in class org.metaqtl. Qtl.QTLTrait 
  
   getCritDelta(String, int[])   - 
Method in class org.metaqtl. MetaQtlResult 
  
   getCriteria()   - 
Static method in class org.metaqtl. EMCriteria 
 Returns an iterator on the criteria
   getCriteria()   - 
Method in class org.metaqtl. MetaQtlResult 
  
   getCriterion(int)   - 
Method in class org.metaqtl. EMCriteria 
 Get the criterion value according to its integer
 code.
   getCriterion(String)   - 
Method in class org.metaqtl. EMCriteria 
  
   getCriterion(int)   - 
Method in class org.metaqtl. EMResult 
  
   getCriterion()   - 
Method in class org.metaqtl. MetaQtlModel 
  
   getCriterionIdx(String)   - 
Static method in class org.metaqtl. EMCriteria 
 Returns the integer code for the criterion which matches
 the given name.
   getCritValues(String, int[])   - 
Method in class org.metaqtl. MetaQtlResult 
  
   getCritWeights(String, int[])   - 
Method in class org.metaqtl. MetaQtlResult 
  
   getCrossDesign(IBioGenome)   - 
Static method in class org.metaqtl.util. GeneticMapProperties 
 This routine returns an object which handle the
 properties of the offspring which have been used  
 to create the genetic map.
   getCrossType()   - 
Method in class org.thalia.bio.entity. CrossPopulation 
  
   getCrossType()   - 
Method in interface org.thalia.bio. IBioCross 
  
   getCSIdx(int, int)   - 
Method in class org.metaqtl. CMarkerSequence 
  
   getDataPoints(int, boolean)   - 
Method in class org.metaqtl. MetaQtlData 
 For a given trait group indice this methods returns
 the data points as a matrix of Double X[0..1][0..n-1]
 where X[0] is the vector of the n position values
 and X[1] is the vector of the n standard deviations
 of the positions.
   getDF()   - 
Method in interface org.metaqtl.numrec. NumericalUtilities.Function 
 Returns the derivative of the function.
   getDF()   - 
Method in interface org.metaqtl.numrec. NumericalUtilities.NRFunction 
 Returns the value of the derivate in x;
   getDist()   - 
Method in class org.metaqtl. TreeNode 
  
   getDistance(int, int, int)   - 
Method in class org.metaqtl. Chromosome 
 This methods returns the distance between the first marker and
 the marker with the given indice using the given mapping context.
   getDistance(int)   - 
Method in class org.metaqtl. Chromosome 
 This methods returns the distance between the first marker and
 the marker with the given indice using the default mapping
 context.
   getDistanceBetween(int, int, int, int)   - 
Method in class org.metaqtl. Chromosome 
 This methods returns the distance between the markers with
 the given indices assuming that  mrkIdxL < mrkIdxR .
   getDistanceBetween(int, int)   - 
Method in class org.metaqtl. Chromosome 
  
   getDistHeterogeneity(int, int[][], Chromosome, Chromosome)   - 
Static method in class org.metaqtl. Chromosome 
  
   getEMEps()   - 
Method in class org.metaqtl.algo. QtlClustAlgorithm 
  
   getEMRanStartNumber()   - 
Method in class org.metaqtl.algo. QtlClustAlgorithm 
  
   getEuclidean()   - 
Method in class org.metaqtl. EMResult 
  
   getF()   - 
Method in interface org.metaqtl.numrec. NumericalUtilities.NRFunction 
 Returns the value of the function in x.
   getFirstCommonMrkIdx(Chromosome, Chromosome)   - 
Static method in class org.metaqtl. Chromosome 
  
   getFunction()   - 
Method in class org.thalia.bio.entity. Ontology 
  
   getFunction()   - 
Method in interface org.thalia.bio. IBioOntology 
 Returns the function of the ontology
   getGeneration()   - 
Method in class org.thalia.bio.entity. Population 
  
   getGeneration()   - 
Method in interface org.thalia.bio. IBioCross 
  
   getGenome()   - 
Method in class org.thalia.bio.entity. LocusContainer 
  
   getGenome()   - 
Method in interface org.thalia.bio. IBioLGroup 
  
   getGraph()   - 
Method in class org.metaqtl. ChromCluster 
  
   getGroup()   - 
Method in class org.thalia.bio.entity. AlleleContainer 
  
   getGroup(String)   - 
Method in class org.thalia.bio.entity. GroupContainer 
  
   getGroup()   - 
Method in class org.thalia.bio.entity. Locus 
  
   getGroup(String)   - 
Method in interface org.thalia.bio. IBioGenome 
  
   getGroup()   - 
Method in interface org.thalia.bio. IBioLocus 
  
   getGroups()   - 
Method in class org.thalia.bio.entity.bean. GeneticMapBean 
  
   getHeight()   - 
Method in class org.metaqtl.graph. ChromAxe 
  
   getHeight()   - 
Method in class org.metaqtl.graph. ChromLayer 
  
   getHeight()   - 
Method in class org.metaqtl.graph. GraphShape 
  
   getHeight()   - 
Method in class org.metaqtl.graph. Layer 
  
   getHeight()   - 
Method in class org.metaqtl.graph. MetaGraph 
  
   getHeight()   - 
Method in class org.metaqtl. TreeNode 
  
   getHeigth()   - 
Method in class org.metaqtl.graph. QtlShape 
  
   getID()   - 
Method in class org.thalia.bio.entity. OntologyTerm 
  
   getID()   - 
Method in interface org.thalia.bio. IBioOntologyTerm 
 Returns the id if the terms.
   getImage(ChromGraph[])   - 
Static method in class org.metaqtl.graph. GraphFactory 
  
   getIndividual(String)   - 
Method in class org.thalia.bio.entity. IndividualContainer 
  
   getIndividual(String)   - 
Method in interface org.thalia.bio. IBioPopulation 
  
   getIndividualNumber()   - 
Method in class org.thalia.bio.entity. IndividualContainer 
  
   getIndividualNumber()   - 
Method in class org.thalia.bio.entity. Population 
  
   getIndividualNumber()   - 
Method in interface org.thalia.bio. IBioPopulation 
  
   getInstance()   - 
Static method in class org.thalia.bio.util. BioEntityNameComparator 
  
   getInstance()   - 
Static method in class org.thalia.bio.util. LocusPositionComparator 
  
   getK()   - 
Method in class org.metaqtl. EMResult 
  
   getK(MetaSimuStat.SimuConfig[], String)   - 
Static method in class org.thalia.metaqtl.main. MetaSimuStat 
  
   getKValues()   - 
Method in class org.metaqtl. MetaQtlResult 
  
   getLabelSide()   - 
Method in class org.metaqtl.graph. ChromShape 
  
   getLayer(double, double)   - 
Method in class org.metaqtl.graph. QtlLayerBuffer 
 Returns a QtlLayer which represent the contents of
 the buffer.
   getLeaf(int)   - 
Method in class org.metaqtl. Tree 
  
   getLeafHeights()   - 
Method in class org.metaqtl. Tree 
  
   getLeafNumber()   - 
Method in class org.metaqtl. Tree 
  
   getLeftCSMarker(int, int)   - 
Method in class org.metaqtl. CMarkerSequence 
 Returns the indices of the marker at the left of the 
 given one.
   getLocus()   - 
Method in class org.thalia.bio.entity.bean. LGroupBean 
  
   getLocus(String)   - 
Method in class org.thalia.bio.entity. LocusContainer 
  
   getLocus()   - 
Method in interface org.thalia.bio. IBioAllele 
  
   getLocus(String)   - 
Method in interface org.thalia.bio. IBioLGroup 
  
   getLocusAdapter()   - 
Static method in class org.thalia.bio.entity. Locus 
  
   getLocusNumber()   - 
Method in class org.thalia.bio.entity. LocusContainer 
  
   getLocusNumber()   - 
Method in interface org.thalia.bio. IBioLGroup 
  
   getLocusType()   - 
Method in class org.thalia.bio.entity. Marker 
  
   getLocusType()   - 
Method in class org.thalia.bio.entity. QTL 
  
   getLocusType()   - 
Method in interface org.thalia.bio. IBioLocus 
  
   getMahalanobis(double[])   - 
Method in class org.metaqtl. EMResult 
  
   getMap(IBioGenome[], String)   - 
Static method in class org.metaqtl.main. MetaMain 
  
   getMapName()   - 
Method in class org.metaqtl. Chromosome 
  
   getMappingCross(IBioGenome)   - 
Static method in class org.metaqtl.util. GeneticMapProperties 
  
   getMappingFunction(IBioGenome)   - 
Static method in class org.metaqtl.util. GeneticMapProperties 
 This methods returns the integer code of the mapping
 function associated to the given genetic map.
   getMappingUnit(IBioGenome)   - 
Static method in class org.metaqtl.util. GeneticMapProperties 
  
   getMarkerDico()   - 
Method in class org.metaqtl. ChromCluster 
  
   getMarkerDico()   - 
Method in class org.metaqtl. MapCluster 
  
   getMarkerIdx(int, int, int)   - 
Method in class org.metaqtl. CMarkerSequence 
  
   getMarkerIdxWithName(String)   - 
Method in class org.metaqtl. Chromosome 
 This method looks if it exists a marker on the chromosome
 with the given name and returns its indice.
   getMarkerIndexByName(String)   - 
Method in class org.metaqtl. ChromCluster 
 For a given marker name this method returns
 the marker index of the marker table if found.
   getMarkerIntervalNumber()   - 
Method in class org.metaqtl. ChromCluster 
 This method returns the total number of marker intervals,
 i.e the sum over chromosomes of the number of marker intervals.
   getMarkerName(String)   - 
Method in class org.metaqtl. ChromCluster 
 This method returns the name of the locus
 from the given name.
   getMarkerName(int)   - 
Method in class org.metaqtl. Chromosome 
 Returns the names of the marker at the i^th position
 on the chromosome.
   getMarkerNameByIndex(int)   - 
Method in class org.metaqtl. ChromCluster 
 This method returns the name of the marker 
 according to this indice.
   getMarkerNumber()   - 
Method in class org.metaqtl. ChromCluster 
 This method returns the number of distinct markers implied
 in this cluster, i.e the size of the marker table.
   getMarkerOccurence(int)   - 
Method in class org.metaqtl. ChromCluster 
 Returns the number of times the marker with
 the indice i has been seen over the
 chromosomes of the cluster.
   getMetaOccurence(IBioLocus)   - 
Static method in class org.metaqtl.util. LocusProperties 
  
   getMetaQtl(String, String, int)   - 
Method in class org.metaqtl. MetaQtlAnalysis 
 Returns the meta Qtl for the given chromosome indice
  cidx , the given trait name  trait 
 and the given number of clusters  k .
   getMetaQtl(int)   - 
Method in class org.metaqtl. MetaQtlResult 
  
   getMetaQtls()   - 
Method in class org.metaqtl. Chromosome 
  
   getModel(String, String)   - 
Method in class org.metaqtl. MetaQtlModel 
  
   getModel(int, int)   - 
Method in class org.metaqtl. MetaQtlModel 
  
   getMrkTickPoint2D(int, boolean)   - 
Method in class org.metaqtl.graph. ChromLayer 
  
   getMrkTickPoint2D(int, boolean)   - 
Method in class org.metaqtl.graph. ChromShape 
  
   getMrkToRm(File)   - 
Static method in class org.metaqtl.main. MetaMain 
  
   getMu(int)   - 
Method in class org.metaqtl. EMResult 
  
   getMu(MetaSimuStat.SimuConfig[])   - 
Static method in class org.thalia.metaqtl.main. MetaSimuStat 
  
   getName()   - 
Method in class org.metaqtl. ChromCluster 
 Returns the name of the cluster.
   getName()   - 
Method in class org.metaqtl. Chromosome 
 Returns the name of the chromosome.
   getName()   - 
Method in class org.metaqtl. Qtl 
 Returns the name of the QTL.
   getName()   - 
Method in class org.metaqtl. TreeNode 
  
   getName()   - 
Method in class org.thalia.bio.entity.bean. BioEntityBean 
  
   getName()   - 
Method in class org.thalia.bio.entity.bean. PropertyBean 
  
   getName()   - 
Method in class org.thalia.bio.entity. BioEntity 
  
   getName()   - 
Method in class org.thalia.bio.entity. BioEntityCollection 
  
   getName()   - 
Method in interface org.thalia.bio. IBioEntity 
 Returns the name of the biological entity
   getNodeHeights()   - 
Method in class org.metaqtl. Tree 
  
   getNodeNumber()   - 
Method in class org.metaqtl. Tree 
  
   getO(MetaSimuStat.SimuConfig[])   - 
Static method in class org.thalia.metaqtl.main. MetaSimuStat 
  
   getOption()   - 
Method in exception org.metaqtl.main. CmdLineParser.IllegalOptionValueException 
  
   getOptionChar()   - 
Method in exception org.metaqtl.main. CmdLineParser.NotFlagException 
  
   getOptionName()   - 
Method in exception org.metaqtl.main. CmdLineParser.UnknownOptionException 
  
   getOptionValue(CmdLineParser.Option)   - 
Method in class org.metaqtl.main. CmdLineParser 
 Equivalent to   getOptionValue(o,
 null)  .
   getOptionValue(CmdLineParser.Option, Object)   - 
Method in class org.metaqtl.main. CmdLineParser 
  
   getOptionValues(CmdLineParser.Option)   - 
Method in class org.metaqtl.main. CmdLineParser 
  
   getPalette(int)   - 
Static method in class org.metaqtl.graph. PaletteColor 
 Returns a palete of colors which should be distinct.
   getPaletteGradient(int, Color, Color)   - 
Static method in class org.metaqtl.graph. PaletteColor 
  
   getParent()   - 
Method in class org.metaqtl. TreeNode 
  
   getParent()   - 
Method in class org.thalia.bio.entity. BioEntity 
  
   getParent()   - 
Method in class org.thalia.bio.entity. BioEntityCollection 
  
   getParent()   - 
Method in interface org.thalia.bio. IBioEntity 
 Returns the entity that handles this entity.
   getParents()   - 
Method in class org.thalia.bio.entity. CrossPopulation 
  
   getParents()   - 
Method in class org.thalia.bio.entity. Individual 
  
   getParents()   - 
Method in class org.thalia.bio.entity. Population 
  
   getParents()   - 
Method in interface org.thalia.bio. IBioCross 
  
   getParents()   - 
Method in interface org.thalia.bio. IBioIndividual 
 Returns the pedigree of the individual
   getParentTerm()   - 
Method in class org.thalia.bio.entity. OntologyTerm 
  
   getParentTerm()   - 
Method in interface org.thalia.bio. IBioOntologyTerm 
 Returns the parent of the term.
   getPartNames()   - 
Method in class org.metaqtl. QtlPartition 
  
   getPedigree()   - 
Method in class org.thalia.bio.entity. Individual 
  
   getPi(int)   - 
Method in class org.metaqtl. EMResult 
  
   getPopulation()   - 
Method in class org.thalia.bio.entity. Individual 
  
   getPopulation()   - 
Method in interface org.thalia.bio. IBioIndividual 
  
   getPosition()   - 
Method in class org.thalia.bio.entity. AlleleContainer 
  
   getPosition()   - 
Method in class org.thalia.bio.entity. Locus 
  
   getPosition()   - 
Method in interface org.thalia.bio. IBioLocus 
  
   getPredict(MetaSimuStat.SimuConfig[], String)   - 
Static method in class org.thalia.metaqtl.main. MetaSimuStat 
  
   getProba()   - 
Method in class org.metaqtl. Chromosome 
  
   getProbaColor(double)   - 
Static method in class org.metaqtl.graph. MetaGraphPar 
  
   getProbaColor(double, double)   - 
Static method in class org.metaqtl.graph. MetaGraphPar 
  
   getProperties()   - 
Method in class org.thalia.bio.entity.bean. BioEntityBean 
  
   getProperties()   - 
Method in class org.thalia.bio.entity. BioEntity 
  
   getProperties()   - 
Method in class org.thalia.bio.entity. BioEntityCollection 
  
   getProperties()   - 
Method in interface org.thalia.bio. IBioEntity 
 Gets the properties of the entity.
   getQtl(int, int)   - 
Method in class org.metaqtl. MetaQtlData 
 Returns the j^th QTL in the i^th cluster.
   getQtlIdx(int)   - 
Method in class org.metaqtl. MetaQtlData 
 Returns the indices of the Qtl for the given
 trait cluster indice  i .
   getQTLInMrkIntProb(int, int, int)   - 
Method in class org.metaqtl. Chromosome 
 This methods returns the probability that the QTL be inside 
 the marker interval with the given right and left marker indices
 and the given QTL indice.
   getQTLInMrkIntProb(int, int, Qtl)   - 
Method in class org.metaqtl. Chromosome 
 This methods returns the probability that the QTL be inside 
 the marker interval with the given right and left marker indices
 and the given QTL.
   getQtlNumber()   - 
Method in class org.metaqtl. Chromosome 
  
   getQtlNumber()   - 
Method in class org.metaqtl. MetaQtlData 
  
   getQtlPartition(Chromosome, IBioOntology)   - 
Static method in class org.metaqtl.main. MetaMain 
  
   getQtlPartition(Chromosome, MetaQtlAnalysis, String, int, char)   - 
Static method in class org.metaqtl.main. MetaMain 
  
   getQtlPartNames()   - 
Method in class org.metaqtl.graph. QtlPartitionLayer 
  
   getQtls()   - 
Method in class org.metaqtl. Chromosome 
 This method returns a copy of the QTL located
 on thic chromosome.
   getQTLSD(Qtl.QTLSupport, int, int)   - 
Static method in class org.metaqtl. Qtl 
 Depending on the CI type this methods returns the  
 the standard deviation of the QTL position using the given
 mapping context.
   getQTLSD(double, Qtl.QTLCross, int)   - 
Static method in class org.metaqtl. Qtl 
 Depending on the r-square parameter and the cross design
 this method returns the standard deviation of the QTL position
 for the given mapping context.
   getQtlTree()   - 
Method in class org.metaqtl. MetaQtlResult 
  
   getRecHeterogeneity(int, int[][], Chromosome, Chromosome)   - 
Static method in class org.metaqtl. Chromosome 
  
   getRemainingArgs()   - 
Method in class org.metaqtl.main. CmdLineParser 
  
   getResources(IResource, IBioEntity[])   - 
Static method in class org.thalia.bio.util. ResourceBioEntityQuery 
  
   getResult()   - 
Method in class org.metaqtl.algo. ClustAlgorithm 
 When the algorithm is finished use this method to get
 the result.
   getResult()   - 
Method in class org.metaqtl.algo. MetaMapAlgorithm 
  
   getResult()   - 
Method in class org.metaqtl.algo. QtlProjAlgorithm 
  
   getResult(String, String)   - 
Method in class org.metaqtl. MetaQtlAnalysis 
 Returns the result for the given trait name or null
 if not found.
   getResult(String)   - 
Method in class org.metaqtl. MetaQtlAnalysis 
 Returns the first result for the given chromosome.
   getResult(int, String)   - 
Method in class org.metaqtl. MetaQtlAnalysis 
 Returns the result for the given trait name or null
 if not found.
   getResults(String)   - 
Method in class org.metaqtl. MetaQtlAnalysis 
  
   getRightCSMarker(int, int)   - 
Method in class org.metaqtl. CMarkerSequence 
  
   getRoot()   - 
Method in class org.metaqtl. Tree 
  
   getRoot()   - 
Method in class org.thalia.bio.entity. Ontology 
  
   getRoot()   - 
Method in interface org.thalia.bio. IBioOntology 
 Returns the root of the ontology tree.
   getSD(int)   - 
Method in class org.metaqtl. EMResult 
  
   getSD1(MetaSimuStat.SimuConfig[])   - 
Static method in class org.thalia.metaqtl.main. MetaSimuStat 
  
   getSD2(MetaSimuStat.SimuConfig[])   - 
Static method in class org.thalia.metaqtl.main. MetaSimuStat 
  
   getSize()   - 
Method in class org.thalia.bio.entity. Population 
 Returns the size of the population.
   getSize()   - 
Method in interface org.thalia.bio. IBioCross 
  
   getSubChromosome(String[])   - 
Method in class org.metaqtl. Chromosome 
  
   getSuboption()   - 
Method in exception org.metaqtl.main. CmdLineParser.UnknownSuboptionException 
  
   getSynonymous(String)   - 
Method in class org.metaqtl. MetaDico 
  
   getTerm(String)   - 
Method in class org.metaqtl. MetaDico 
  
   getTerm(IBioOntologyTerm)   - 
Method in class org.thalia.bio.entity. Ontology 
  
   getTerm(String)   - 
Method in class org.thalia.bio.entity. Ontology 
  
   getTerm(IBioOntologyTerm)   - 
Method in interface org.thalia.bio. IBioOntology 
 Returns if exists the term in the ontology which 
 matches the given one.
   getTerm(String)   - 
Method in interface org.thalia.bio. IBioOntology 
 This method looks into the ontology if there is 
 a term with the given name and returns it.
   getTerms()   - 
Method in class org.metaqtl. MetaDico 
  
   getTraitClusterName(int)   - 
Method in class org.metaqtl. MetaQtlData 
  
   getTraitClusterNames()   - 
Method in class org.metaqtl. MetaQtlData 
 Returns the names of the trait clusters.
   getTraitClusterNumber()   - 
Method in class org.metaqtl. MetaQtlData 
 Returns the number of trait groups for this data set as
 defined by the clustering method.
   getTraitClusterSize(int)   - 
Method in class org.metaqtl. MetaQtlData 
 Returns the size of the trait cluster i.
   getTraitIdx(int, String)   - 
Method in class org.metaqtl. MetaQtlAnalysis 
  
   getTraitName()   - 
Method in class org.metaqtl. MetaQtlResult 
 Returns the name of the trait cluster for this result.
   getTraitNames(String)   - 
Method in class org.metaqtl. MetaQtlModel 
  
   getTraitNames(int)   - 
Method in class org.metaqtl. MetaQtlModel 
  
   getType()   - 
Method in class org.thalia.bio.entity. AlleleContainer 
  
   getType()   - 
Method in class org.thalia.bio.entity. BioEntity 
  
   getType()   - 
Method in class org.thalia.bio.entity. BioEntityCollection 
  
   getType()   - 
Method in class org.thalia.bio.entity. CrossPopulation 
  
   getType()   - 
Method in class org.thalia.bio.entity. GeneticMap 
  
   getType()   - 
Method in class org.thalia.bio.entity. GroupContainer 
  
   getType()   - 
Method in class org.thalia.bio.entity. Individual 
  
   getType()   - 
Method in class org.thalia.bio.entity. IndividualContainer 
  
   getType()   - 
Method in class org.thalia.bio.entity. LGroup 
  
   getType()   - 
Method in class org.thalia.bio.entity. LocusContainer 
  
   getType()   - 
Method in class org.thalia.bio.entity. Marker 
  
   getType()   - 
Method in class org.thalia.bio.entity. Ontology 
  
   getType()   - 
Method in class org.thalia.bio.entity. OntologyTerm 
  
   getType()   - 
Method in class org.thalia.bio.entity. Population 
  
   getType()   - 
Method in class org.thalia.bio.entity. QTL 
  
   getType()   - 
Method in interface org.thalia.bio. IBioEntity 
 There are 2 main class of entities.
   getType()   - 
Method in interface org.thalia.bio. IBioLocus 
  
   getType()   - 
Method in class org.thalia.bio.util. EstimatedLocusPosition 
  
   getType()   - 
Method in interface org.thalia.bio.util. ILGroupPosition 
  
   getType()   - 
Method in class org.thalia.bio.util. LGroupPosition 
  
   getType()   - 
Method in class org.thalia.bio.util. StaticLocusPosition 
  
   getUSD(EMResult, int, int, String)   - 
Method in class org.metaqtl. MetaQtlResult 
  
   getValue()   - 
Method in exception org.metaqtl.main. CmdLineParser.IllegalOptionValueException 
  
   getValue(String, Locale)   - 
Method in class org.metaqtl.main. CmdLineParser.Option 
  
   getValue()   - 
Method in class org.thalia.bio.entity.bean. PropertyBean 
  
   getVLabels()   - 
Method in class org.metaqtl. ChromCluster.ClusterGraph 
  
   getWidth()   - 
Method in class org.metaqtl.graph. ChromLayer 
  
   getWidth()   - 
Method in class org.metaqtl.graph. GraphShape 
  
   getWidth()   - 
Method in class org.metaqtl.graph. Layer 
  
   getWidth()   - 
Method in class org.metaqtl.graph. MetaGraph 
  
   getWidth()   - 
Method in class org.metaqtl.graph. QtlShape 
  
   getWorkAmount()   - 
Method in class org.metaqtl.algo. MetaAlgorithm 
  
   getWorkAmount()   - 
Method in interface org.metaqtl. IMetaAlgorithm 
 Returns the amount of the work to do.
   getWorkProgress()   - 
Method in class org.metaqtl.algo. MetaAlgorithm 
  
   getWorkProgress()   - 
Method in interface org.metaqtl. IMetaAlgorithm 
 Returns the amount of work which have already
 been done.
   getX()   - 
Method in class org.metaqtl.graph. AnchoredElement 
  
   getX()   - 
Method in class org.metaqtl.graph. ChromAxe 
  
   getX()   - 
Method in class org.metaqtl. TreeNode 
  
   getX(MetaSimuStat.SimuConfig[])   - 
Static method in class org.thalia.metaqtl.main. MetaSimuStat 
  
   getXBestPred()   - 
Method in class org.metaqtl. EMResult 
  
   getXChrom()   - 
Method in class org.metaqtl.graph. ChromShape 
  
   getXPred()   - 
Method in class org.metaqtl. EMResult 
  
   getXPredIdx(int)   - 
Method in class org.metaqtl. EMResult 
  
   getY()   - 
Method in class org.metaqtl.graph. AnchoredElement 
  
   getY()   - 
Method in class org.metaqtl. TreeNode 
  
   getYDistance(QtlShape, QtlShape)   - 
Static method in class org.metaqtl.graph. QtlShape 
 Computes the distance between two Qtl shapes assuming
 along the same axe Y (i.e the shapes have same X).
   getYMax()   - 
Method in class org.metaqtl.graph. ChromAxe 
  
   getYMax()   - 
Method in class org.metaqtl.graph. ChromLayer 
  
   getYMax()   - 
Method in class org.metaqtl.graph. ChromShape 
  
   getYMax()   - 
Method in class org.metaqtl.graph. GraphShape 
  
   getYMax()   - 
Method in class org.metaqtl.graph. Layer 
  
   getYMax()   - 
Method in class org.metaqtl.graph. QtlLayer 
  
   getYMax()   - 
Method in class org.metaqtl.graph. QtlPartitionLayer 
  
   getYMax()   - 
Method in class org.metaqtl.graph. QtlShape 
  
   getYMax()   - 
Method in class org.metaqtl.graph. QtlTreeLayer 
  
   getYMin()   - 
Method in class org.metaqtl.graph. ChromAxe 
  
   getYMin()   - 
Method in class org.metaqtl.graph. ChromLayer 
  
   getYMin()   - 
Method in class org.metaqtl.graph. ChromShape 
  
   getYMin()   - 
Method in class org.metaqtl.graph. GraphShape 
  
   getYMin()   - 
Method in class org.metaqtl.graph. Layer 
  
   getYMin()   - 
Method in class org.metaqtl.graph. QtlLayer 
  
   getYMin()   - 
Method in class org.metaqtl.graph. QtlPartitionLayer 
  
   getYMin()   - 
Method in class org.metaqtl.graph. QtlShape 
  
   getYMin()   - 
Method in class org.metaqtl.graph. QtlTreeLayer 
  
   getZ(int)   - 
Method in class org.metaqtl. EMResult 
  
   GraphFactory   - Class in  org.metaqtl.graph      GraphFactory()   - 
Constructor for class org.metaqtl.graph. GraphFactory 
  
   GraphShape   - Class in  org.metaqtl.graph      GraphShape()   - 
Constructor for class org.metaqtl.graph. GraphShape 
  
   group   - 
Variable in class org.metaqtl. Qtl.QTLTrait 
 The name of the group to which
 the trait belongs.
   GroupContainer   - Class in  org.thalia.bio.entity  Class Description Here   GroupContainer()   - 
Constructor for class org.thalia.bio.entity. GroupContainer 
  
   GroupContainer(String, IBioEntity)   - 
Constructor for class org.thalia.bio.entity. GroupContainer 
  
   groupNumber()   - 
Method in class org.thalia.bio.entity. GroupContainer 
  
   groupNumber()   - 
Method in interface org.thalia.bio. IBioGenome 
  
   groups   - 
Variable in class org.thalia.bio.entity.bean. GeneticMapBean 
  
   groups()   - 
Method in class org.thalia.bio.entity. GroupContainer 
  
   groups()   - 
Method in interface org.thalia.bio. IBioGenome 
  
   gser(double, double)   - 
Static method in class org.metaqtl.numrec. NumericalUtilities 
  
 
 


 
   
  
 
 
 
   
 
   
          Overview     
        Package   
        Class   
        Use   
          Tree     
          Deprecated     
       Index    
          Help     
   
 
 
  
 
 
 

 
  
   PREV LETTER   
   NEXT LETTER    
  
    FRAMES     
   NO FRAMES     
  
    All Classes  ');
  }
  //-->
 
 
    All Classes  
 


  
 
 
  
 

 A   B   C   D   E   F   G   H   I   K   L   M   N   O   P   Q   R   S   T   U   V   W   X   Y   Z
